# When minority language persistence is not enough: the decline of foraging knowledge in German- and Ladin-speaking Alpine communities of Northern Italy

**DOI:** 10.1186/s13002-026-00906-4

**Published:** 2026-05-09

**Authors:** Irfan Ullah, Mousaab Alrhmoun, Syed Waseem Gillani, Julia Prakofjewa, Lei Zhang, Giulia Mattalia, Hawraz Ibrahim M. Amin, Cheikh Yebouk, Faisal Moola, Paolo Corvo, Renata Sõukand, Raivo Kalle, Naji Sulaiman, Andrea Pieroni

**Affiliations:** 1 Department of Molecular Wood Biotechnology and Technical Mycology, Georg- August University of Göttingen, Büsgenweg 2, 37077 Göttingen, Germany; 2 University of Gastronomic Sciences, Piazza Vittorio Emanuele II 9, Pollenzo, 12042 Italy; 3 Faculty of Agricultural, Environmental and Food Sciences, Free University of Bolzano, Piazza Università 5, Bolzano, 39100 Italy; 4 Department of Plant Sciences, Quaid-I-Azam University, Islamabad, Pakistan; 5 Department of Environmental Sciences, Informatics, and Statistics, Ca’ Foscari University of Venice, Via Torino 155, Venice, 30174 Italy; 6 Institute of Vegetable and Flower Research, Chinese Academy of Agricultural Sciences, Beijing, China; 7 Facultat de Farmàcia i Ciències de l’Alimentació –Institut de Recerca de la Biodiversitat (IRBio), Laboratori de Botànica–Unitat Associada CSIC, Universitat de Barcelona, Barcelona, 08028 Catalonia Spain; 8 Department of Chemistry, University of Pavia, Viale Taramelli 12, Pavia, 27100 Italy; 9 Department of Pharmacy, Tishk International University, Erbil, 44001 Iraq; 10 Laboratory of Plant Biodiversity and Natural Resource Development, University of Nouakchott, BP 880, Nouakchott, Mauritania; 11 Department of Geography, Environment and Geomatics, University of Guelph, Guelph, Canada; 12 Estonian Literary Museum, Vanemuise 42, Tartu, 51003 Estonia; 13 Department of Medical Analysis, Tishk International University, Erbil, 44001 Iraq

**Keywords:** Wild food plants, Ethnobotany, Ethnomycology, Local ecological knowledge, Socio-ecological change, Lusern, Mocheni, Ladins

## Abstract

Local ecological knowledge is an essential element of the Alpine biocultural diversity and is often linked to minority languages. However, its continuity does not necessarily correspond to the persistence of these languages. This study investigates how knowledge of wild plants (and mushrooms) is transmitted and transformed among three minority-language communities in the Italian Alps: Cimbrian, Mòcheno, and Ladin speakers. Ethnobotanical data were collected through structured interviews between 2022 and 2025 and compared with historical records from the 1980s documenting the use of wild plants for food, medicinal purposes, and herbal teas. The results reveal a marked decline in traditional plant-related practices despite the continued use and institutional support of minority languages. Plants once central to household herbal repertoires, such as *Achillea millefolium* L., *Artemisia absinthium* L., and *Sambucus nigra* L. which are now marginal or abandoned. However, a limited set of herbal teas remains culturally significant. Conversely, mushroom foraging and the gathering of wild fruits remain comparatively resilient, supported mainly by communal land-use institutions (*usi civici*) that foster ecological engagement and intergenerational learning. Overall, the study highlights a paradox of biocultural resilience: linguistic vitality alone does not safeguard embodied ecological knowledge. Sustaining Alpine cultural and environmental heritage, therefore, requires integrated approaches that address language, environment, governance, and socio-economic change together.

## Introduction

Minority language vitality and local ecological knowledge are key components of Alpine biocultural diversity [7, 30]. LEK encompasses the adaptive knowledge that local communities develop through lived interactions with their environment, while minority languages embody cultural identities and vernacular traditions often marginalised within broader national frameworks [25, 36, 45]. Both are increasingly threatened by globalisation, cultural homogenization, urbanisation, and biodiversity overexploitation [12, 46]. Yet, both remain vital to understanding how communities adapt to social and environmental change.

LEK is inherently dynamic and functions as an adaptive management system that enables communities to respond to ecological uncertainty and socioecological transformation [19, 24, 44]. The resilience of LEK refers to the ability of local communities to draw upon accumulated, place-based knowledge to address challenges such as climate change, biodiversity loss, resource depletion, and sociopolitical disruption [22]. Similarly, the resilience of minority languages reflects their capacity to maintain cultural significance and intergenerational transmission despite external pressures. The decline of minority languages often results from the dominance of national languages in education, media, and administration [12, 46]. Consequently, revitalisation efforts are essential to preserving both linguistic heritage and the knowledge systems embedded within these languages.

Globally, the resilience of LEK and minority languages often appears interconnected. The persistence of Indigenous or minority languages is frequently tied to the maintenance of traditional and customary practices, especially those related to environmental stewardship that are communicated and sustained through language [15, 37]. Thus, minority groups that retain their languages are often better able to preserve ecological knowledge systems essential for adapting to environmental change. Conversely, communities that sustain traditional ecological practices tend to maintain the local vocabularies and conceptual frameworks that reflect their lived environments [1, 13].

Minority languages frequently encode specific terminology for local flora, fauna, and ecological processes, encapsulating centuries of environmental observation and classification. As Maffi analysed two decades ago [27], the decline of linguistic diversity often parallels the erosion of biodiversity and LEK. Over the past two decades, growing attention has been devoted to linguistic diversity as a key component of cultural rights and biocultural heritage.

Local ecological knowledge (LEK) is central to understanding interactions between communities and their environments. It supports resource management, cultural practices, and adaptation to socio-environmental change. Aswani et al., [8] demonstrate that LEK underpins community-based management of natural environments. Language serves as the principal medium for transmitting such knowledge through customs, beliefs, and cultural practices related to the stewardship of nature [28, 31]. Collectively, these studies affirm that sustaining linguistic diversity is inseparable from sustaining environmental stewardship. Safeguarding both requires more than policy reform: it demands integrating local knowledge into education, protecting linguistic rights, and promoting minority languages in public and cultural life. Such combined efforts by policymakers, educators, and communities can strengthen both cultural identity and ecological resilience, contributing to more inclusive pathways toward sustainability.

Evidence from diverse regions suggests that ecological practices and language are often interconnected [2, 3, 6, 7, 32, 39, 52].

Successful strategies of resilience often rely on intergenerational transmission, where elder community members teach younger generations both linguistic and ecological competencies [19]. Language revitalisation programs that integrate ecological knowledge demonstrate how intertwined these systems can be. Nevertheless, the relationship between linguistic and environmental resilience is not always reciprocal. LEK can persist in communities where language transmission has weakened, and conversely, linguistic revitalisation may not guarantee the continuity of ecological practices. Similar patterns of long-term erosion in herbal knowledge have been documented in other Alpine regions, where diachronic comparisons reveal substantial losses over recent decades despite cultural continuity, largely attributable to socio-economic and livelihood transformations [53].

Empirical examples illustrate this complexity. In Pakistan’s Kohat district, LEK persisted among communities who had shifted mainly to dominant regional languages, demonstrating that ecological knowledge can survive linguistic loss [17, 50].

Similarly, Mendoza et al., [29] showed that fishing communities may retain ecological management practices even when minority languages have declined, suggesting that economic dependence on natural resources can sustain LEK independently of language vitality [18, 29]. These cases underscore that while linguistic and ecological resilience are often correlated, they do not necessarily depend on one another. Each can persist under particular sociocultural and economic conditions. Economic and structural transformations such as globalisation and market integration may further reshape these dynamics. As communities adapt to new livelihoods, dominant languages may replace local vernaculars, while LEK evolves to incorporate novel practices and terminologies aligned with emerging economic opportunities [56]. Consequently, linguistic and ecological systems may follow divergent trajectories, revealing both resilience and vulnerability within biocultural heritage.

This study examines the persistence and transformation of LEK related to wild food plants and mushrooms among Cimbrian, Mòcheno, and Ladin communities in Northern Italy. The research had two main objectives:

1. To document the LEK related to WFPMs that are still foraged or remembered in the German-speaking communities of Luserna and the Bernstol (Mòcheni Valley) and in the Ladin-speaking areas of the lower Fassa Valley, Cadore, and Ampezzo.
2. To compare this contemporary knowledge with ethnobotanical data recorded in the same regions during the 1980s, in order to identify processes of persistence, erosion, and transformation.

The analysis focuses on diachronic change and cross-cultural variation within differing socio-economic contexts. This study does not assess linguistic vitality quantitatively. Instead, it focuses on ethnobotanical knowledge, while language is considered as a contextual factor.

## Materials and methods

### The study area and field study

**Fig. 1:**
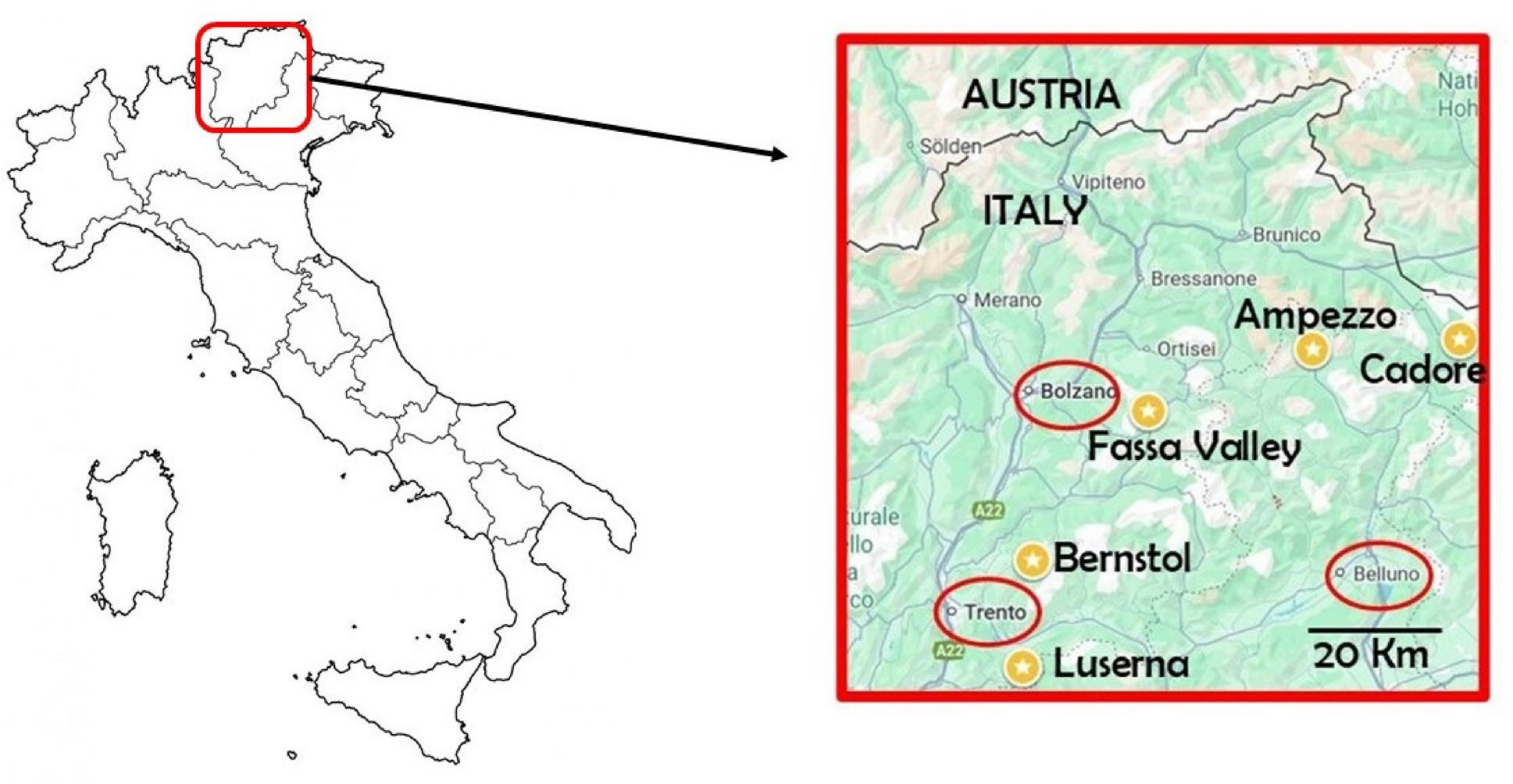
The study villages and areas (marked with an asterisk); the most inhabited centres of the surrounding regions are circled in red

Field ethnobotanical research was conducted between 2022 and 2025 across five Alpine areas in northeastern Italy (Fig. 1), focusing on three minority-language groups of Bavarian- and Ladin-speaking heritage.

**Fig. 2:**
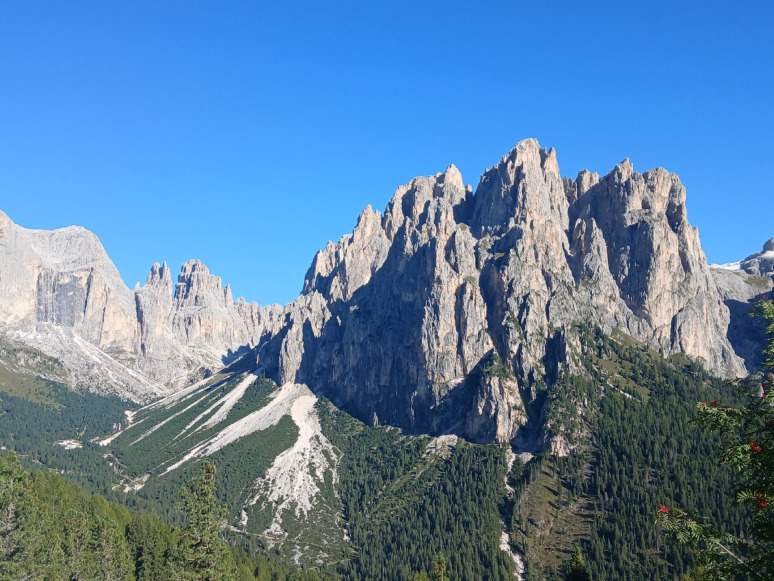

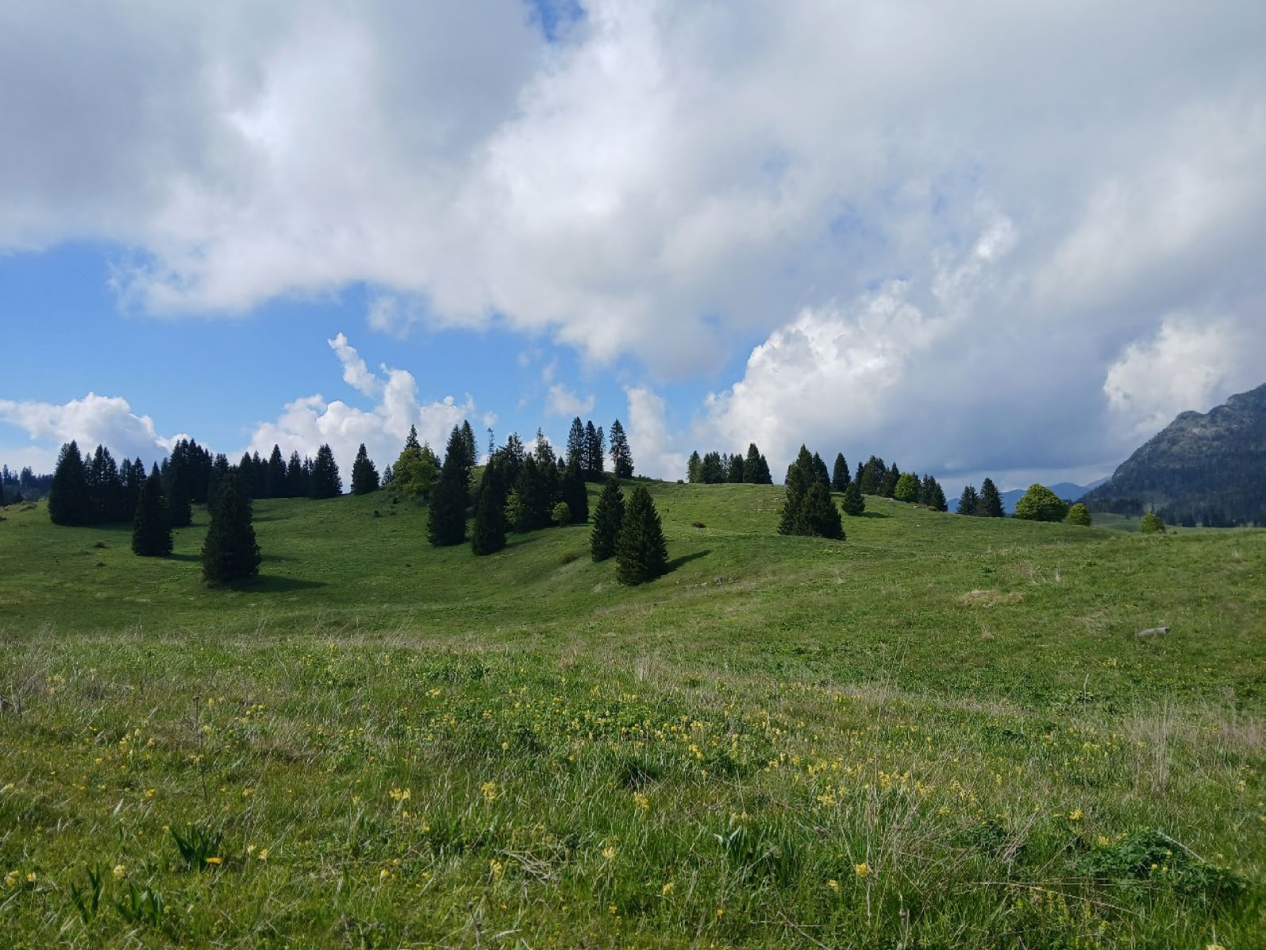

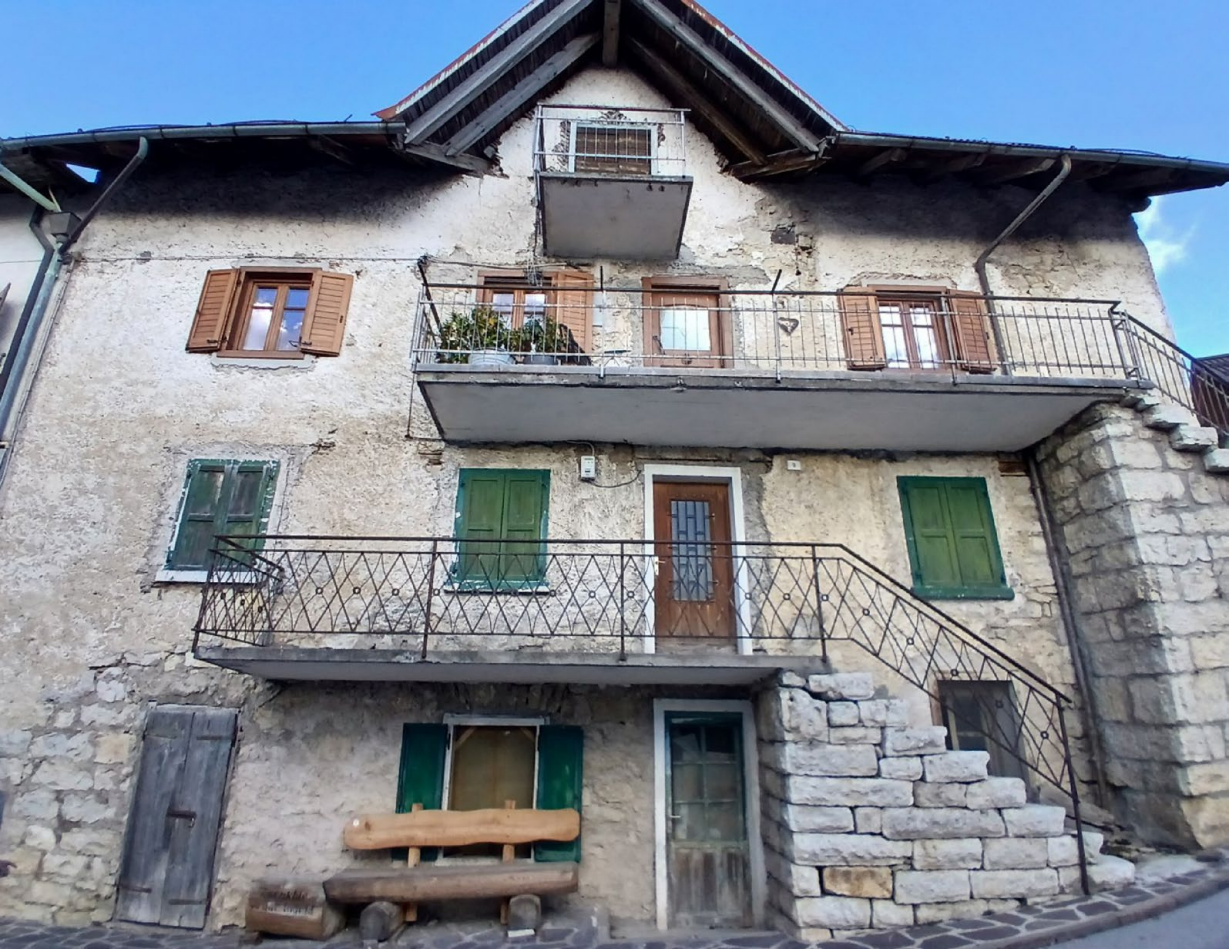
A few pictures of the Alpine landscapes around Lusern/Luserna and Vigo di Fassa, and an old house in Lusern. Photo credit: A.P

The first group represents speakers of Cimbrian (ISO 639 code: cim), a Bavarian German variety spoken in Lusern/Luserna (Trento Province, 1,333 m.a.s.l.). The second group speaks Mòcheno (mhn), another Bavarian German variety, represented by the communities of Bernstol/Valle dei Mòcheni (Palù del Fersina, 1,360 m.a.s.l.; Fierozzo, 1,116 m.a.s.l., Trento Province). The third group includes Ladin (lld) speakers from three localities: Fassa (Vigo di Fassa and adjacent summer hamlets, 1,382 m.a.s.l., Trento Province), Ampezzano (Cortina d’Ampezzo, Belluno Province, 1,224 m.a.s.l.), and Cadorino (Pieve di Cadore, Belluno Province, 878 m.a.s.l.). Additional nearby villages around Cortina and Pieve were also included. A few pictures of the Alpine landscapes around the field study are illustrated in Fig. 2.

All study sites are located within typical Alpine environments historically shaped by an intensive agro-pastoral economy [33]. Over the last few decades, this has shifted markedly toward tourism, though with distinct trajectories across communities. Lusern, Bernstol, and Cadore, owing to their relative geographic peripherality, experience moderate to heavy tourism, while Ampezzo and the Fassa Valley have been strongly affected by over-tourism, a phenomenon increasingly observed in other Alpine regions.

A total of 73 semi-structured interviews were conducted in Italian with farmers, shepherds, elderly residents, restaurant owners, librarians, and representatives of local ethnographic museums and municipalities. Informants were selected through purposive and snowball sampling, targeting individuals with experience in farming, foraging, or local ecological practices. The sample included participants from the following localities: Lusern (*n* = 18; mean age 65; 9 male, 9 female), Bernstol/Mòcheni Valley (*n* = 18; mean age 68; 10 male, 8 female; distributed across Palù del Fersina, Frassilongo, and San Felice), Fassa Valley (*n* = 15; mean age 67; 10 male, 5 female; Vigo and Pozza di Fassa), and Cadore/Ampezzo (*n* = 22; mean age 63; 12 male, 10 female; Pieve, Calalzo, Auronzo, and Cortina). Interviews were conducted primarily in Italian as a shared lingua franca, but in German in several cases within German-speaking communities. All participants were bilingual in the minority language and Italian, and frequently reported using their minority language in daily life. The use of Italian as an interview language therefore did not preclude access to local ecological knowledge, as plant names, practices, and narratives were often expressed bilingually during the interviews. The discussions focused on current and remembered foraging practices for wild food plants and mushrooms. In Cadore and Ampezzo, interviews concentrated on wild vegetables and mushrooms. At the same time, in the Fassa Valley and the German-speaking areas, the survey also included wild fruits, seasoning herbs, and plants used in herbal teas and homemade liqueurs.

Each folk taxon was identified using several approaches. When possible, fresh plant specimens were collected and identified using Flora d’Italia and its online portal (https://dryades.units.it/floritaly/index.php). When specimens were unavailable, identification was based on comparisons with earlier collections from the same areas or on detailed descriptions provided by interviewees regarding local names, ecology, and morphology. In cases of uncertainty, interviewees were shown detailed digital photographs or botanical drawings to confirm identification.

Botanical and mycological nomenclature follows World Flora Online (https://www.WorldFloraOnline.org/) and Index Fungorum (https://www.indexfungorum.org/), with family assignments according to the Angiosperm Phylogeny Website (https://www.mobot.org/mobot/research/apweb/). Collected herbarium specimens are stored in the Herbarium of Ca’ Foscari University of Venice (UVV) under accession numbers UVVITGR01–25. Ethical approval was granted by the Ethics Committee of the University of Gastronomic Sciences, and all fieldwork complied with the Code of Ethics of the International Society of Ethnobiology (https://www.ethnobiology.net/what-we-do/core-programs/ise-ethics-pro gram/ code-of-ethics/).

### Data analysis

The recorded uses of wild plants for food and medicinal purposes (including herbal teas and liqueurs) were systematically compared with previous ethnobotanical and ethnomedical studies conducted in the same Alpine regions. For the Cimbrian-speaking community of Lusern, no prior ethnobotanical documentation was available, while for the Bernstoler Mòcheno community, contemporary results were compared with data collected in the early 1980s by botanist Elsa Maria Cappelletti and collaborators [14]. For the Ladin-speaking communities of the Fassa Valley and Cadore, present-day data were contrasted with studies from the 1980s conducted by Cesare Poppi and Helma Holzknecht, which combined original field research and reviews of ethnographic sources from the same period [43]. Although historical and contemporary datasets differ in sampling intensity and research focus, the comparison remains valid for identifying broad patterns of persistence, erosion, and transformation in locally salient taxa.

All ethnobotanical records were organised and coded in Microsoft Excel to facilitate systematic cross-cultural and temporal analyses. Each folk taxon was annotated with its scientific and local name(s), plant part(s) used, type of preparation, present and past use, and its primary functional category (culinary or medicinal). This coding enabled the identification of taxa that have persisted across generations, as well as those that have been lost, transformed, or newly adopted in local repertoires. Venn diagrams were generated in Excel to visualise overlaps and unique taxa among linguistic groups and across time periods. Differences in sampling intensity, research focus, and historical data availability between past and present datasets may affect comparability. Therefore, the diachronic analysis should be interpreted as indicative of general trends rather than exact quantitative equivalence. The Use Value (UV) was calculated to assess the relative importance of each taxon based on frequency of citation [35]. The Informant Consensus Factor (ICF) was used to evaluate the degree of agreement among informants for specific use categories [55]. The Shannon diversity index (H′) was applied to estimate the diversity of ethnobotanical knowledge within each community [51]. The Jaccard similarity index was used to compare similarities in taxa between communities and across time periods [23].

## Results

### Overview of recorded taxa and uses

We identified 71 wild food plants and mushroom taxa still recorded among the Cimbrian and Mòcheno speakers (Table 1). The results for the Ladin-speaking communities are presented separately below (Table 2). The overall Use Value (UV) ranged from 0.05 to 0.65, with the highest values attributed to *Urtica dioica* L., *Taraxacum venustum* Dahlst., *Vaccinium myrtillus* L., and *Boletus* spp., reflecting their strong cultural salience and frequent use in daily life. The Shannon diversity index (H′ = 2.83) indicates moderate ethnobotanical diversity across both communities. However, the Informant Consensus Factor (ICF) varied across use categories, being highest for digestive and respiratory treatments (0.78 and 0.74, respectively), suggesting cohesive shared knowledge in these domains.

Nevertheless, the use of several taxa now survives only in memory, as confirmed by comparison with historical data collected from the same Mòcheno community several decades ago. Notably, some taxa indicated by the absence of current folk names in Table 1 are no longer part of daily practice, which likely reflects reduced current use or recall rather than direct linguistic erosion.

**Table 1:** Local foraged wild food plants and mushrooms recorded among the studied German-speaking enclaves in Trentino, compared with data collected a few decades ago in Bernstol; local phytonyms of non-German origin are reported in italics; local names in Cimbrian are reported without label, whereas those in Mòcheno are indicated by (M)

Taxon	Family	Local Cimbrian or Mòcheno (M) recorded folk name(s)	Used parts	Food and/or herbal use(s) (treated diseases)	Recorded past Bernstoler use (x); remembered Cimbrian use (+)
*Achillea millefolium* L.	Asteraceae	Rossokum	Leaves	Tea: depurative, for hypertension, digestive, menstrual pains	x+
*Agaricus campestris* L.	Agaricaceae	Kröas, *Prataioli*, *Buacce* (M), *Prateri* (M)	Fruiting body	Cooked	
*Alchemilla xanthochlora* Rothm.	Rosaceae	-	Leaves	Tea: hypertension, dysmenorrhoea	x
*Allium schoenoprasum* L. ITGR03	Amaryllidaceae	*Sivel*	Aerial parts	Seasoning	
*Arctostaphylos uva-ursi* (L.) Spreng.	Ericaceae	Billeöpfele, *Uva ursina* (M)	Leaves	Tea: diuretic	x
*Aria edulis* (Willd.) M.Roem.	Rosaceae	Melpeirpumma	Fruits	Snack, seasoning grappa	+
*Armillaria mellea* (Vahl) P. Kumm.	Physalacriaceae	*Chiodini*, Stocksbem	Fruiting body	Cooked	
*Arnica montana* L.	Asteraceae	-	Flowering tops	Tea: sedative	x
*Artemisia absinthium* L.	Asteraceae	Bermat, Wermat	Aerial parts	Tea, seasoning grappa and wine: digestive, emetic	x
*Artemisia vulgaris* L.	Asteraceae	-	Leaves	Tea: digestive	x
*Athyrium filix-femina* (L.) Roth	Athyriaceae	-	Aerial parts	Tea: diuretic	x
*Berberis vulgaris* L.	Berberidaceae	Spiboirmarn, Spidorn	Fruits	Seasoning grappa	+
*Betula pendula* Roth.	Betulaceae	Birch (M)	Leaves; wood	Tea: arthritis; burned for smoking meat	x+
*Boletus* spp.	Boletaceae	*Brigalde*,* Brise* (M)	Fruiting body	Cooked; in the past, boiled, strained, and preserved in oil, pepper, and garlic	
*Calendula officinalis* L.	Asteraceae	-	Leaves and flowers	Tea: digestive	x
*Calluna vulgaris* (L.) Hill	Ericaceae	-	Flowers	Tea: diuretic	x
*Calocybe gambosa* (Fr.) Donk	Lyophyllaceae	*Fungo di saetta*	Fruiting body	Cooked	
*Cantharellus cibarius* Fr.	Hydnaceae	*Finferli*, Finferling (M)	Fruiting body	Cooked	
*Craterellus lutescens* (Fr.) Fr.	Hydnaceae	Finferle	Fruiting body	Cooked	
*Carlina acaulis* L.	Asteraceae	Khesedorn	Flower receptacles	Snack	+
*Carum carvi* L.	Apiaceae	Kim, Kum, Kare (M), Kumin (M), Kamin (M)	Fruits	Seasoning; Tea: digestive, cough	x
*Cetraria islandica* (L.) Ach.	Parmeliaceae	*Lichene*, Rach von hekkela	Thallus	Tea: cough, cold	x+
*Chenopodium album* L.	Amaranthaceae	*Farinei*	Leaves	Cooked	+
*Chenopodium bonus-henricus* (L.) Rchb. ITGR21	Amaranthaceae	Bilsgekraut, Hummerkraut, Hummegekraüt, Gekraut, Scheisskraut (M), Shpinat (M)	Leaves	Cooked with rice, polenta, and eggs	
*Clavariadelphus pistillaris* (L.) Donk	Clavariadelphaceae	*Fungo d’Ercole*	Fruiting body	Cooked	
*Clitocybe nebularis* (Batsch) P. Kumm.	Clitocybaceae	*Moreti bianchi*,* Sbim*	Fruiting body	Cooked	
*Collybia nuda* (Bull.) Z.M. He & Zhu L. Yang	Clitocybaceae	*Moreti del vin*	Fruiting body	Cooked	
*Corylus avellana* L. ITGR10	Betulaceae	Hasenuss, Nussel (M)	Seeds	Snack	
*Crataegus monogyna* Jacq.	Rosaceae	-	Flowers	Tea: sedative	
*Equisetum sylvaticum* L.	Equisetaceae	-	Aerial parts	Tea: cough, digestive	x
*Fagus sylvatica* L.	Fagaceae	Puach	Fruits	Snack	+
*Fragaria vesca* L. ITGR11	Rosaceae	Ruatpeeren, Peren, Eapar (M), Eipper (M), Japar (M), Ertpeeren (M)	Fruits	Raw, Jams, Tea: depurative	x
*Galium odoratum* (L.) Scop.	Rubiaceae	*Sperula*	Aerial parts	Seasoning grappa	
*Gentiana acaulis* L.	Gentianaceae	-	Roots	Seasoning grappa: digestive	x
*Juniperus communis* L.	Cupressaceae	Kronebit, Kronebit (M)	Galbuls; branches	Seasoning meat and grappa; burned to smoke meat	
*Lactarius deliciosus* (L.) Gray ITGR08	Russulaceae	Roatlin, *Fungo de pin*,* Fungo del sangue*, Finferguerblut (M)	Fruiting body	Cooked	
*Lamium album* L. ITGR23.	Lamiaceae	Essel (M)	Flowers	Sucked as a snack	x
*Larix decidua* (L.) Mill.	Pinaceae	Lerch, Larch (M)	Shoots; resin	Seasoning grappa; sucked	
*Lycoperdon* spp.	Lycoperdaceae	*Sloffe*	Fruiting body	Cooked	
*Macrolepiota procera* (Scop.) Singer	Agaricaceae	*Ombrella*,* Ombrella* (M)	Fruiting body	Cooked	
*Malva neglecta* Wallr.	Malvaceae	Pappel, *Malvia* (M)	Leaves and roots	Tea: depurative, stomachache, cough	x
*Matricaria chamomilla* L.and *Matricaria discoidea* DC. ITGR20	Asteraceae	Kamamila, Komomille (M)	Flowering tops	Tea: digestive, dysmenorrhoea, cough	x
*Mentha* spp.	Lamiaceae	-	Aerial parts	Tea: digestive, stomach-ache, fever, diarrhoea	x
*Morchella* sp.	Morchellaceae	Möech, Möer	Fruiting body	Cooked	
*Nasturtium officinale* R.Br.	Brassicaceae	-	Leaves	Tea: diuretic	x
*Nepeta cataria* L.	Lamiaceae	-	Leaves	Seasoning grappa: digestive	x
*Persicaria bistorta* (L.) Samp.	Polygonaceae	Zeimpulluma	Leaves	Cooked	+
*Picea abies* (L.) H.Karst.	Pinaceae	Tänn	Shoots, resin	Tea: cough; resin mixed with sugar: cough	x
*Pinus cembra* L. ITGR13	Pinaceae	Zirm (M)	Cones and shoots; resin	Seasoning grappa; sucked	
*Pinus mugo* Turra ITGR12	Pinaceae	*Pin*, Tschurschla (cones)	Cones and shoots; resin	Seasoning grappa; tea: cough	x
*Primula veris* L. ITGR02	Primulaceae	Tscutscharla, Tuscherla	Flowers	Tea: cough, sedative; sucked	x
*Ramaria aurea* (Schaeff.) Quél.	Gomphaceae	*Chate di galina*	Fruiting body	Cooked	
*Ribes alpinum* L.	Grossulariaceae	Heirnpeeren, *Uospinela*	Fruits	Raw, jams	+
*Rosa acicularis* Lindl.	Rosaceae	-	Petals	Tea: hoarseness	x
*Rosa canina* L.	Rosaceae	Schneakagen, Skizkageln	Fruits	Tea; cough; jams	+
*Rubus idaeus* L. ITGR19	Rosaceae	Hennepeeren, Himper (M), Empar (M)	Fruits	Raw, jams	
*Rumex acetosa* L. (ITGR16) and *R. acetosella* L.	Polygonaceae	Sauerrampf (M)	Leaves	Snack; salads	
*Rumex alpinus* L.	Polygonaceae	Vlatsch (M)	Leaves	Cooked	+
*Russula cyanoxantha* (Schaeff) Fr.	Russulaceae	*Russule*	Fruiting body	Cooked	
*Russula virescens* (Schaeff.) Fr.	Russulaceae	*Verdoni*,* Verdoni* (M)	Fruiting body	Cooked	
*Sambucus nigra* L. ITGR14	Viburnaceae	Holar	Flowers, fruits, bark	Syrups, jams; tea: cold, cough; ingested	x
*Sambucus racemosa* L.	Viburnaceae	Roat holar, Hollaflieder (M)	Flowers; fruits	Tea: cough; syrup (flowers or fruits)	+
*Silene vulgaris* (Moench) Garcke ITGR22	Caryophyllaceae	*Sgrisoi*, Chernendle	Leaves	Cooked	
*Sorbus aucuparia* L. ITGR09	Rosaceae	Pashtura, Peerboim (M)	Fruits	Seasoning grappa	+
*Tanacetum vulgare* L.	Asteraceae	Sgrigeich honkost	Leaves and flowers	Tea: diuretic, vermifuge; frittata	x
*Taraxacum venustum* Dahlst. ITGR05, ITGR06	Asteraceae	Radik, Pipakona, Huntenzen (M), Hendenzen (M)	Leaves, flowers, roots	Salads, soups; preserved with sugar (flowers); tea: depurative, digestive, rheumatisms	x
*Thymus pulegioides* L.	Lamiaceae	-	Aerial parts	Tea: asthma	x
*Urtica dioica* L. ITGR17	Urticaceae	Essel, Esseln (M)	Leaves	Cooked, tea: depurative	x
*Vaccinium myrtillus* L.	Ericaceae	Schbartzpeeren, Schwarzbar (M), Schwarzwer (M), Schworzwar (M)	Fruits and aerial parts	Raw, jams, tea: diabetes	x
*Vaccinium vitis-idaea* L.	Ericaceae	Billeöpfele, Granten (M), Sborzperg (M)	Fruits	Jams; seasoning grappa	
*Valeriana officinalis* L. ITGR25	Caprifoliaceae	-	Leaves	Tea: sedative	x

### Cross-cultural analysis of wild food plants and mushrooms

**Fig. 3:**
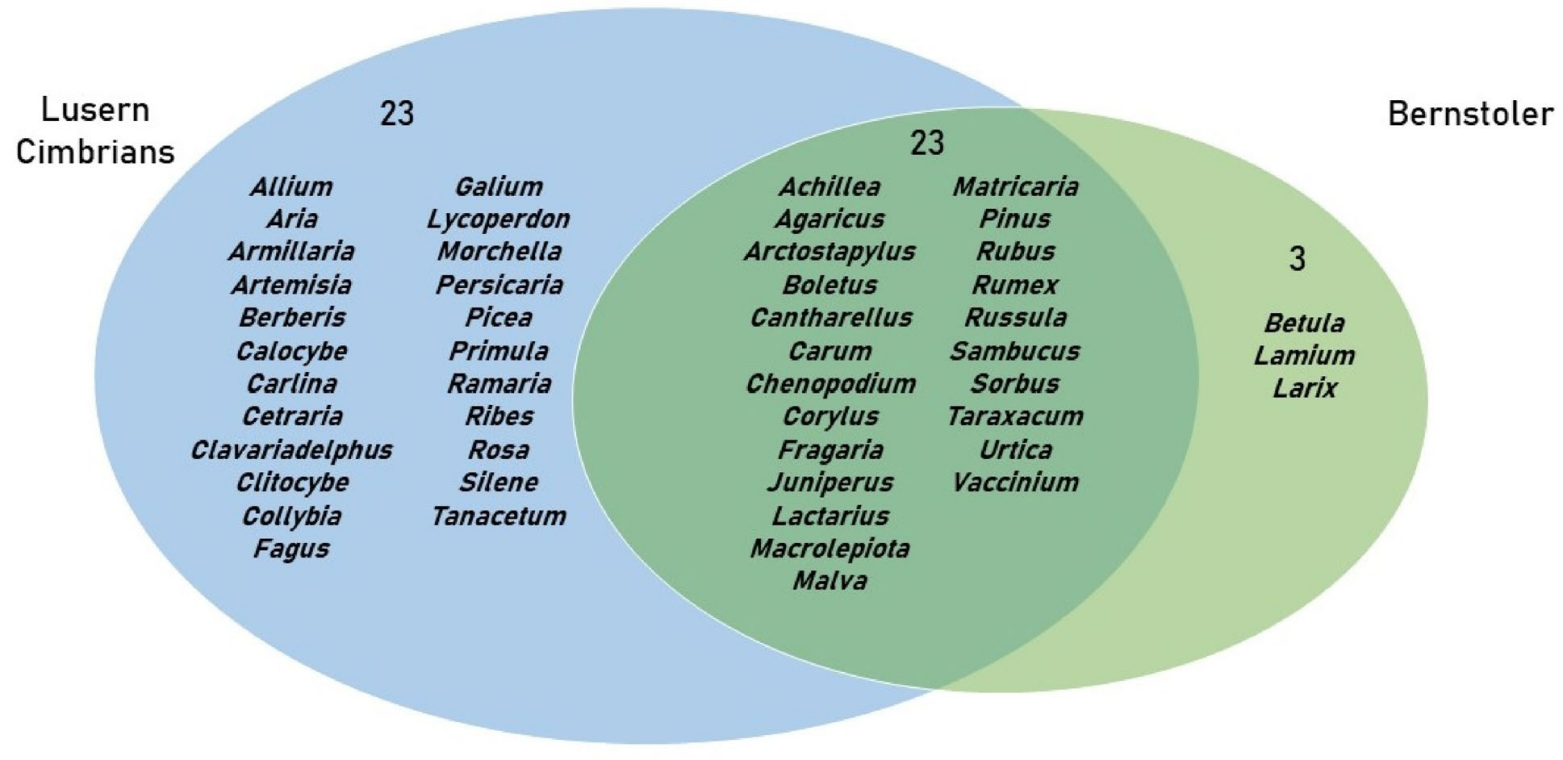
A Venn diagram reflecting the quoted Lusern Cimbrian vs. Bernstoler wild food plant and fungal genera

The comparison of wild plant repertoires between the Cimbrian and Mòcheno communities reveals both shared knowledge and distinct differences, reflecting the interplay between linguistic vitality and local ecological practices (Fig. 3). The Jaccard similarity index between the two communities is 0.41, indicating moderate overlap but substantial differentiation. The Cimbrian community demonstrates a broader range of taxa, particularly forest and meadow species, and a higher ethnobotanical diversity (H′ = 2.9) than the Mòcheno repertoire (H′ = 2.4). In contrast, the Mòcheno knowledge appears more restricted and characterised by lower Cultural Importance Index (CI) scores, concentrated around a few taxa such as Betula pendula, Larix decidua, and Lamium album. In both communities, wild vegetables, fruits, and mushrooms remain part of current foraging practices, though local uses of several taxa are recalled only from memory.

Among the taxa exclusively mentioned by Cimbrians, *Berberis vulgaris*,* Carlina acaulis*,* Persicaria bistorta*, and *Tanacetum vulgare* reflect archaic Alpine foraging patterns. The data suggest that the Cimbrian community retains more resilient LEK, while the Mòcheno group has undergone greater knowledge loss, likely related to reduced engagement with agro-pastoral practices rather than a clear loss of linguistic knowledge.

### Temporal changes in the bernstoler herbal foraging

**Fig. 4:**
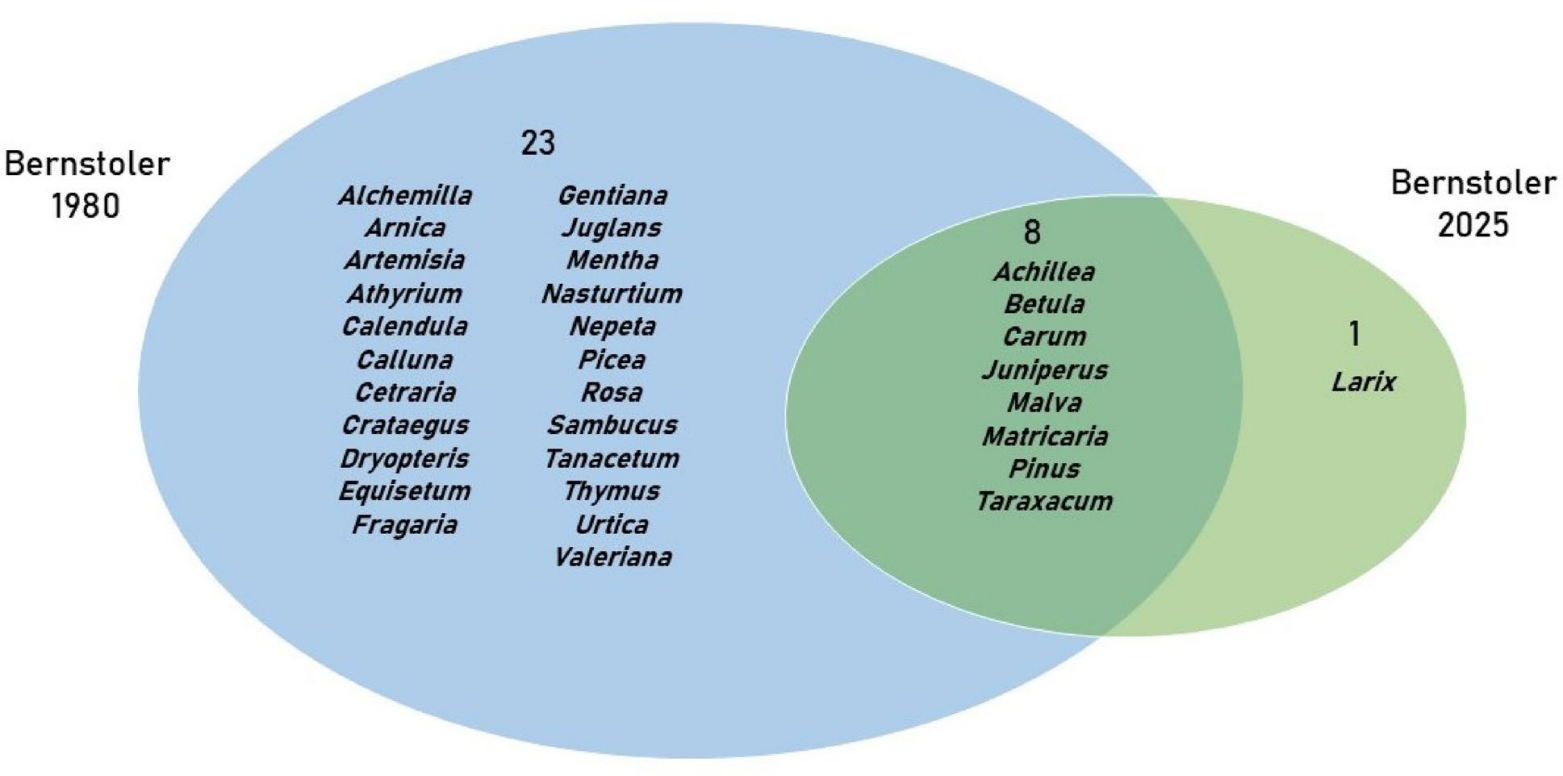
Temporal comparison of herbal plant knowledge in the Bernstoler community, showing species maintained, lost, and newly recorded herbal genera between 1980 and 2025. The comparison has been conducted by considering only the herbal uses of wild plants, as the dataset from the 1980s included only medicinal plants

A diachronic comparison between the present dataset (2025) and ethnobotanical records collected in the early 1980s reveals a substantial 65% decline in the number of wild herbal taxa known or used within the Mòcheno-speaking community (Fig. 4). While 23 genera were reported in the 1980s, only eight remain in current or remembered use. It should be noted that the historical dataset primarily focused on medicinal plants, and fungal uses were not systematically recorded. Therefore, any interpretation of temporal trends in fungal knowledge should be considered qualitative and indicative rather than directly comparable across time.

The Jaccard similarity index (past vs. present) was 0.35, indicating that less than half of the taxa previously known have persisted over the past four decades. Species exhibiting the highest Fidelity Levels (FL > 75%), such as *Achillea millefolium*, *Artemisia absinthium*, and *Urtica dioica* continue to serve as key ingredients in herbal teas and home remedies, demonstrating the stability of a small “core repertoire” of culturally emblematic taxa.

In contrast, many species formerly used in infusions and liquors (*Equisetum sylvaticum*, *Gentiana acaulis*, *Hypericum perforatum*, *Thymus serpyllum*) are now recalled only as “plants used by grandparents.” This reflects a profound generational discontinuity in both practical use and linguistic transmission.

The overall Informant Consensus Factor (ICF) for medicinal plant use categories declined from 0.81 in the historical dataset to 0.58 in the current one, suggesting reduced intra-community agreement and a weakening of shared ethnobotanical knowledge. The erosion of LEK thus appears closely tied to broader socio-economic transformations, particularly the collapse of small-scale farming and the expansion of the tourism-based economy, which have significantly diminished the experiential contexts in which such knowledge was once maintained.

### LEK related to wild food plants and mushrooms of the Ladin speakers

We identified 83 wild food plants and mushroom taxa still recorded among the three Ladin-speaking communities (Table 2). The overall Use Value (UV) was slightly higher than in the Germanic enclaves (mean UV = 0.48), and the Shannon index (H′ = 3.15) indicates higher ethnobotanical diversity, consistent with the ecological and cultural richness of Ladin valleys.

Key taxa such as *Artemisia absinthium*, *Gentiana lutea*, *Sambucus nigra*, and *Taraxacum venustum* retain both high Use Value (UV > 0.6) and Cultural Importance Index (CI > 1.2), attesting to their persistent symbolic and practical relevance.

A comparison with ethnographic records from the 1980s (in Fassa and Gardena Valleys) revealed that approximately 42% of previously cited taxa have disappeared from active use, while foraged wild vegetables, fruits, and mushrooms remain robustly practised [42].

**Table 2:** Recorded local uses of wild food plants and mushrooms among Ladins (in our surveys 2022–2025 and in the pre-existing survey

Taxon	Family	Recorded local names(s)	Used parts	Food and/or herbal use(s)	Fassa Valley 2025 (Our data)	Cadore and Ampezzo 2022 (Our data)	Fassa Valley 1980s (Poppe 1989)	Gardena Valley 1980s Holzknecht, [21]
*Abies alba* Mill.	Pinaceae	-	Youngs hoots	Tea: anti-flu				X
*Achillea millefolium* L.	Asteraceae	Millefiori, F	Aerial parts	Tea: emmenagogue			x	x
*Alchemilla* spp.	Rosaceae	-	Aerial parts	Tea: emmenagogue				x
*Allium schoenoprasum* L.	Amaryllidaceae	Sevoia, C	Aerial parts	Seasoning		x		
*Allium ursinum* L.	Amaryllidaceae	-	Aerial parts	Freshly consumed, an anti-haemorrhagic				x
*Amanita caesarea* (Scop.) Pers.	Amanitaceae	Ovoli, C	Fruiting body	Cooked		x		
*Arctostaphylos uva-ursi* (L.) Spreng.	Ericaceae	Uva ursiva, F	Fruits	Jams	xp			
*Artemisia absinthium* L.	Asteraceae	Assenzio, F; Sent, F	Aerial parts	Macerate in grappa and tea: digestive, anti-fever, anti-stomach-ache	x		x	x
*Artemisia genipi* Stechm.	Asteraceae	Edelraut, F	Aerial parts	Tea: anti-headaches			x	
*Asparagus acutifolius* L.	Asparagaceae	Sapris, C	Young shoots	Frittata		x		
*Betula pendula* Roth.	Betulaceae	-	Aerial parts	Tea: diuretic, anti-rheumatic				x
*Boletus* spp.	Betulaceae	Brusa, F, Porcino, C	Fruiting body	Cooked	x	x		
*Borago officinalis* L.	Boraginaceae	-	Aerial parts	Tea: cardiotonic				x
*Calocybe gambosa (*Fr.) Donk	Lyophyllaceae	Prugnolo, C	Fruiting body	Cooked		x		
*Cantharellus cibarius* Fr.	Hydnaceae	Finferlo, CF	Fruiting body	Cooked	x	x		
*Capsella bursa-pastoris* Medik.	Brassicaceae	Ciabianc, F	Aerial parts	Tea: anti-hypertensive, diuretic, emmenagogue	xp			x
*Carlina acaulis* L.	Asteraceae	Formela, F; Spinaces dela Madonna, G	Flower receptacles	Snack; tea: anti-helminthic	x			x
*Carum carvi* L.	Apiaceae	Ciarie, C; Ciaril, F	Fruits	Seasoning; tea: digestive, anti-stomach-aches	xp		x	
*Centaurium erythraea* Rafn.	Gentianaceae	-	Aerial parts	Tea: anti-fever, hepato-protective				x
*Cetraria islandica* (L.) Ach.		Lichene bianco, F; Muschie bianch, F	Thallus	Decoction in milk: anti-flu, anti-tussive, anti-asthmatic, anti-tuberculosis, anti-stomachache	xp		x	x
*Chenopodium bonus-henricus* L.	Amaranthaceae	Amaite, F; Giambaite, F; Giamaiti, F; Grassola, C	Leaves	Cooked	x	x		
*Cicerbita alpina* (L.) Wallr.	Asteraceae	Radicio de konte, C	Young aerial parts	Salads, cooked		x		
*Collybia nuda* (Bull.) Z.M. He & Zhu L. Yang	Clitocybaceae	Fungo viola, F	Fruiting body	Cooked	x			
*Cortinarius caperatus* (Pers.) Fr.	Cortinariaceae	Cortinario, F	Fruiting body	Cooked	xp			
*Crataegus* spp.	Rosaceae	-	Aerial parts	Tea: cardiotonic				x
*Craterellus cornucopioides* (L.) Pers.	Hydnaceae	Trombetta da morto, C	Fruiting body	Cooked	x	x		
*Cyclocybe cylindracea* (DC.) Vizzini & Angelini	Tubariaceae	Pioppini, C	Fruiting body	Cooked		x		
*Cynodon dactylon* (L.) Pers.	Poaceae	Agram, F	Whole plant	Tea: diuretic, anti-cystitis			x	
*Cypripedium calceolus* L.	Orchidaceae	Zoppelle della Madonna, F	Flowers	Snack	xp			
*Equisetum arvense* L.	Equisetaceae	Peceloc de cian, F	Aerial parts	Tea: anti-cystitis			x	
*Erica arborea* L.	Ericaceae	-	Young shoots	Tea: anti-tussive, anti-rheumatic				x
*Fragaria vesca* L.	Rosaceae	Fragola, F	Fruits	Snack, also as anti-anaemia				x
*Fumaria officinalis* L.	Papaveraceae	-	Aerial parts	Tea: anti-fever				x
*Galium verum* L.	Rubiaceae	-	Aerial parts	Tea: anti-goitre				x
*Gentiana lutea* L.	Gentianaceae	Anziana, C; Gensiana, F	Roots	Macerate in grappa or tea: digestive, anti-diarrhoeal, anti-fever, anti-helminthic, anti-fainting	xp	x	x	x
*Gymnadenia nigra* (L.) Rchb.f.	Orchidaceae	Fiore del sangue, F; Negritella, F; Sangon, F	Flowering tops	Tea: anti-hypertensive	xp		x	
*Humulus lupulus* L.	Cannabaceae	Bruscandol, C	Young shoots	Frittata		x		
*Hypericum perforatum* L.	Hypericaceae	-	Flowering tops	Tea: hepato-protective, emmenagogue				x
*Inula helenium* L.	Asteraceae	-	Flowering tops	Tea: hepato-protective				x
*Juniperus communis* L.	Cupressaceae	Geneivr, F; Zineivr, F	Galbules	Tea and seasoned grappa: anti-fever, anti-gout, anti-rheumatic	x			x
*Lactarius deliciosus* (L.) Gray	Russulaceae	Sangoni, F	Fruiting body	Cooked	x			
*Lamium album* L.	Lamiaceae	-	Aerial parts	Tea: emmenoague				x
*Lamium purpureum* L.	Lamiaceae	-	Flowers	Snack	xp			
*Larix decidua* (L.) Mill.	Pinaceae	Lars, F; Lerch, F	Cones, resin	Seasoning grappa; tea (re): anti-haemorrhagic	xp		x	
*Leontopodium nivale* (Ten.) A.Huet ex Hand.-Mazz	Asteraceae	Stela alpina, F	Flowering tops	Macerate in grappa: digestive; tea: anti-dysmenorrhoeal	xp		x	
*Macrolepiota procera* (Scop.) Singer	Agaricaceae	Mazza di tamburo, CF	Fruiting body	Cooked	x	x		
*Malva sylvestris* L.	Malvaceae	Melvia, F	Aerial parts	Tea: anti-sore throats, anti-tussive, cardiotonic, anti-tuberculosis, emmenagogue			x	
*Matricaria recutita* L.	Asteraceae	Camomilla, F	Flowering tops	Tea or decoction in milk: anti-fever, anti-headaches, tranquillising, anti-masles, anti-mumps, emmenagogue (also in filled pasta), anti-tussive, anti-stomachache	x		x	x
*Melissa officinalis* L.	Lamiaceae	-	Aerial parts	Tea: cardiotonic				x
*Morchella* spp.	Morchellaceae	Spugnola, C	Fruiting body	Cooked		x		
*Paeonia officinalis* L.	Paeoniaceae	-	Seeds	Consumed as an anti-epileptic and against fears				x
*Peucedanum ostruthium* (L.) W.D.J.Koch	Apiaceae	Imperatoria, F	Aerial parts and roots	Seasoning; macerate in grappa: digestive	x			
*Picea abies* (L.) H.Karst.	Pinaceae	-	Young shoots	Tea: anti-tussive, anti-flu				x
*Pinus cembra* L.	Pinaceae	Zirm, F	Young shoots, cones, kernels	Seasoning grappa; tea: anti-flue, anti-tuberculosis; (kernels) snacks; jams (laxative)	x		x	x
*Pinus mugo* Turra.	Pinaceae	Barancio, C	Young shoots, cones	Seasoning grappa; tea: anti-flue, anti-tussive		x		x
*Plantago* spp.	Plantaginaceae	Plantane, F	Aerial parts	Syrup: anti-tussive; tea: anti-asthmatic, anti-rheumatic, emmenagogue	x			x
*Populus alba* L.	Salicaceae	-	Shoots	Ingested as a hepato-protective				x
*Potentilla alba* L.	Rosaceae	-	Leaves	Enolate: anti-epileptic				x
*Prunella vulgaris* L.	Lamiaceae	-	Roots	Consumed as an anti-epileptic				x
*Pulmonaria officinalis* L.	Boraginaceae	-	Aerial parts	Tea: anti-asthmatic, anti-flu, anti-tussive				x
*Ramaria botrytis* (Pers.) Bourdot	Gomphaceae	Fuadine, F	Fruiting body	Cooked	xp			
*Ribes* spp.	Grossulariaceae	Ribes, F	Fruits	Snack, also as an anti-anaemia				x
*Rosa canina* L.	Rosaceae	Baca rossa, F	Pseudofruits	Tea; ingested as a diuretic	xp			x
*Rubus idaeus* L.	Rosaceae	Ampilie, F	Fruits	Raw, jams: anti-anaemia	x			x
*Rumex alpinus* L.	Polygonaceae	Pilon, F	Leaves	Boiled	xp			
*Russula virescens* (Schaeff.) Fr.	Russulaceae	Verdone, C	Fruiting body	Cooked		x		
*Salix* spp.	Salicaceae	-	Bark	Tea: anti-fever				x
*Sambucus nigra* L.	Viburnaceae	Sambughei, C; Sauc, F	Flowers and fruits	Tea: anti-flu, anti-fever, anti-tussive; seasoning grappa (fr); jams (sometimes with apples and plums, also consumed as anti-rheumatic and laxative; fr); fruit wine: diuretic	x	x	x	x
*Sanguisorba major* Hill.	Rosaceae	-	Aerial parts	Tea: anti-asthmatic				x
*Saxifraga* sp.	Saxifragaceae	-	Roots or fresh juice	Ingested as an anti-hydrophobic				x
*Silene vulgaris* (Moench) Garcke	Caryophyllaceae	Sgrisolon, F; S-ciupeto, C	Young shoots	Cooked	x	x		
*Silybum marianum* (L.) Gaertn.	Asteraceae	Spis, F	Aerial parts and fruits	Tea: digestive	xp			
*Stellaria media* (L.) Vill.	Caryophyllaceae	Jentivel, F	Aerial parts	Tea: anti-stomach aches			x	
*Taraxacum venustum* Dahlst.	Asteraceae	Radicio, C; Zuchoria, F	Leaves, roots	Salads, cooked; tea: digestive, anti-fever, liver diseases, anti-rheumatic	x	x	x	x
*Thymus serpyllum* L.	Lamiaceae	Erba de la sopina, F	Aerial parts	Tea: anti-sore throats, tranquillising			x	
*Tilia* spp.	Malvaceae	-	Flowers	Tea: anti-flu				x
*Tricholoma terreum*	Tricholomataceae	Morette, C	Fruiting body	Cooked		x		
*Tussilago farfara* L.	Asteraceae	-	Aerial parts	Tea: anti-asthmatic, anti-rheumatic				x
*Urtica dioica* L.	Urticaceae	Urtìe, F; Autria, C	Leaves	Cooked; tea: anti-anaemia, anti-rheumatic, laxative	x	x		x
*Vaccinium myrtillus* L.	Ericaceae	Ciaveise, F	Fruits, Leaves	Snack, also as anti-anaemia, anti-diarrhoeal; tea (le): anti-diabetic; tea and jams (fr): anti-diarrhoeal, diuretic	x		x	x
*Vaccinium vitis-idaea* L.	Ericaceae	Garnete, F	Fruits	Jams; tea (anti-cystitis)	x		x	
*Verbascum* spp.	Scrophulariaceae	-	Aerial parts	Tea: anti-asthmatic, anti-flu				x
*Viscum album* L.	Santalaceae	-	Aerial parts	Tea: cardiotonic				x

C: local name recorded in Cadore and Ampezzo areas; F: folk name recorded in Fassa Valley; G: folk name recorded in Gardena Valley (1980s); p: recorded in the 2025 Fassa Valley field study as past use only

### Diachronic and cross-spatial Analysis of wild food plants and mushrooms among ladins

The diachronic comparison clearly shows the resilience of “core” medicinal taxa (*Artemisia*, *Matricaria*, *Sambucus*, *Taraxacum*, *Vaccinium*) and a sharp decline in the use of common synanthropic herbs (*Achillea*, *Malva*, *Cynodon*, *Stellaria*).

**Fig. 5:**
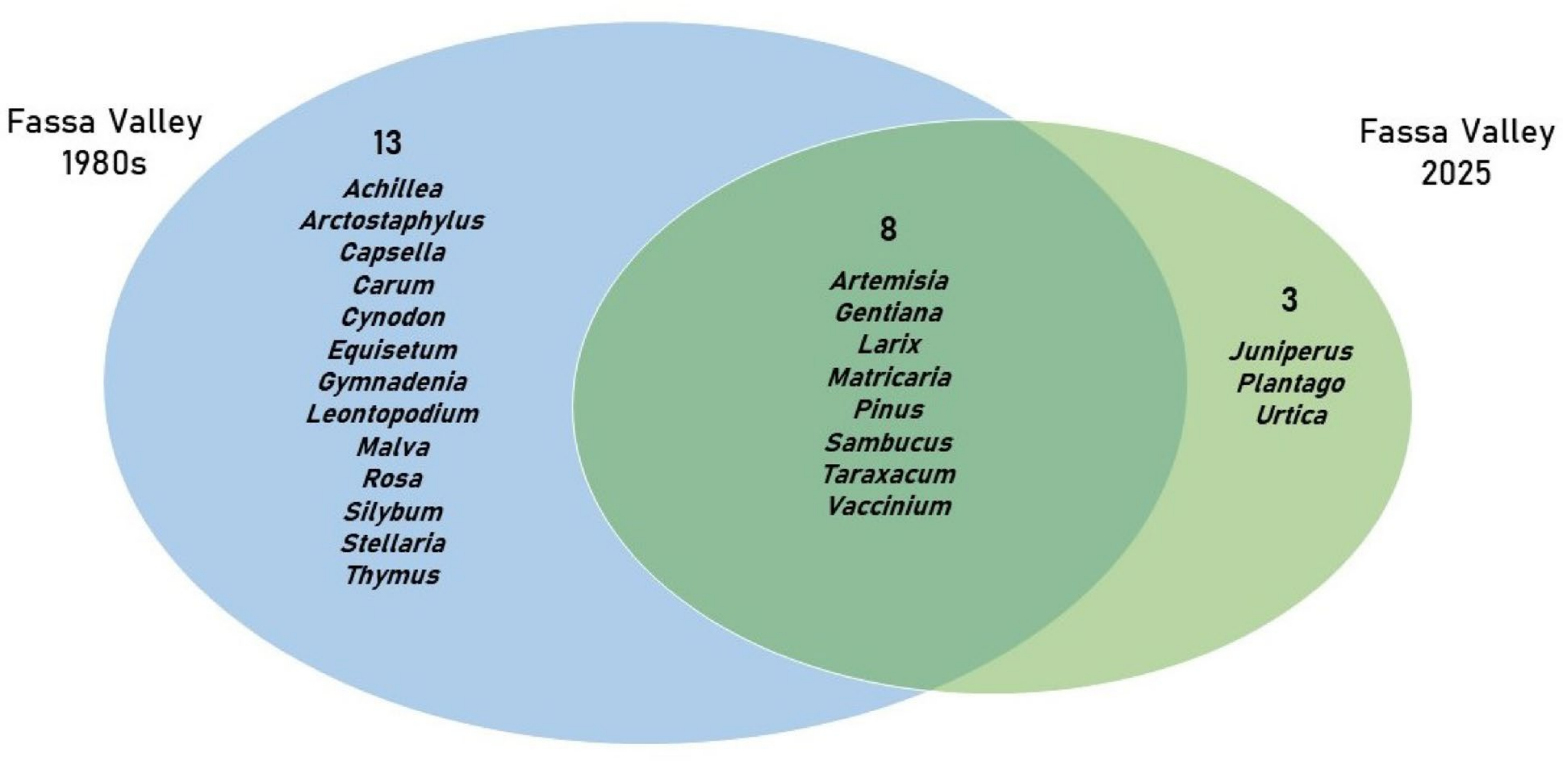
Venn Diagram of the genera reported as locally used in herbal practices in the Fassa Valley in 2025 and in the 1980s

**Fig. 6:**
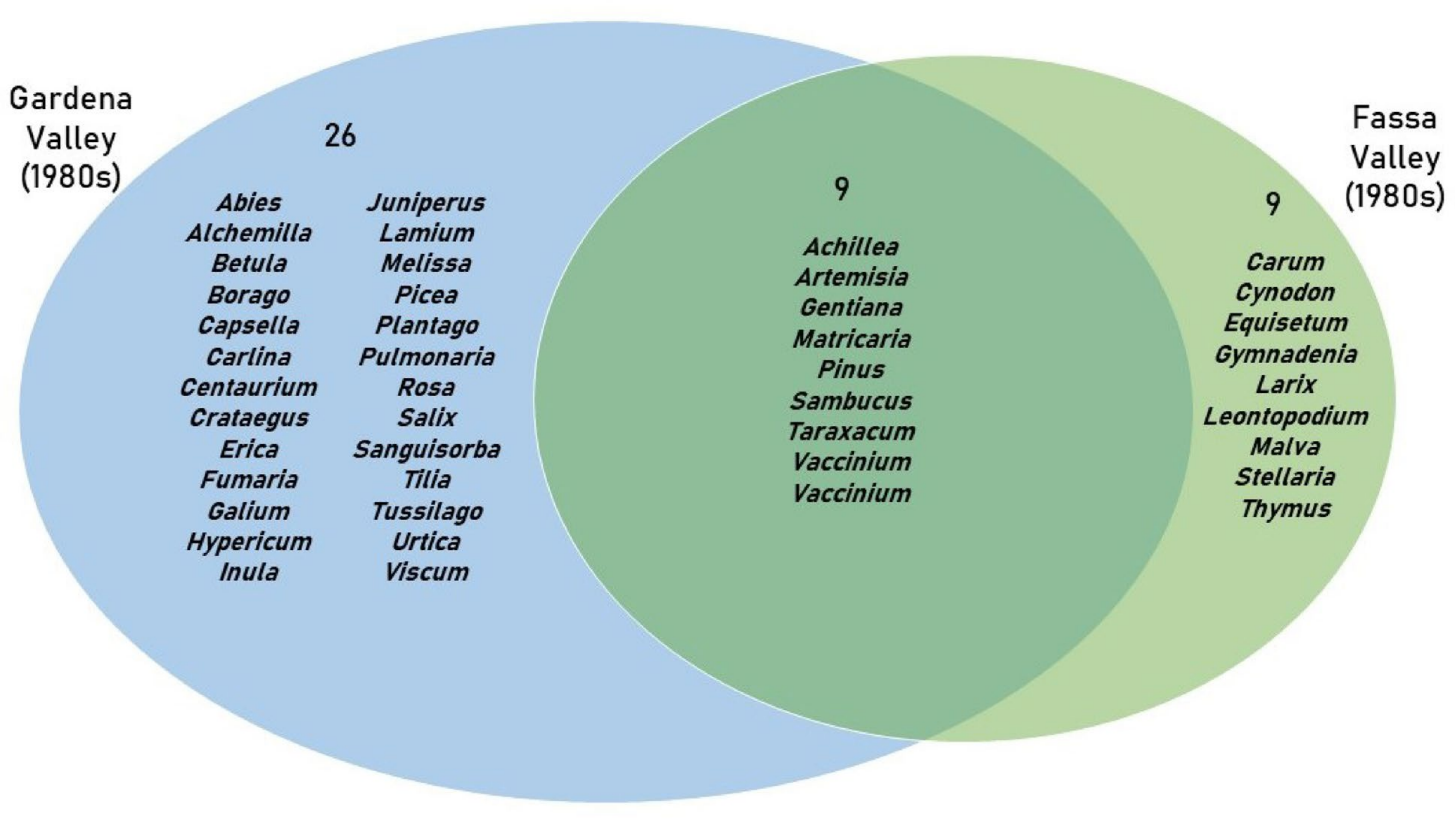
Venn Diagram showing the overlaps between the Ladin wild plant genera reported as locally used in herbal practices a few decades ago in the Fassa and Gardena valleys. In this diagram, we compared the 1980s surveys of the Gardena and Fassa Valleys regarding wild herbal teas and liqueurs only

**Fig. 7:**
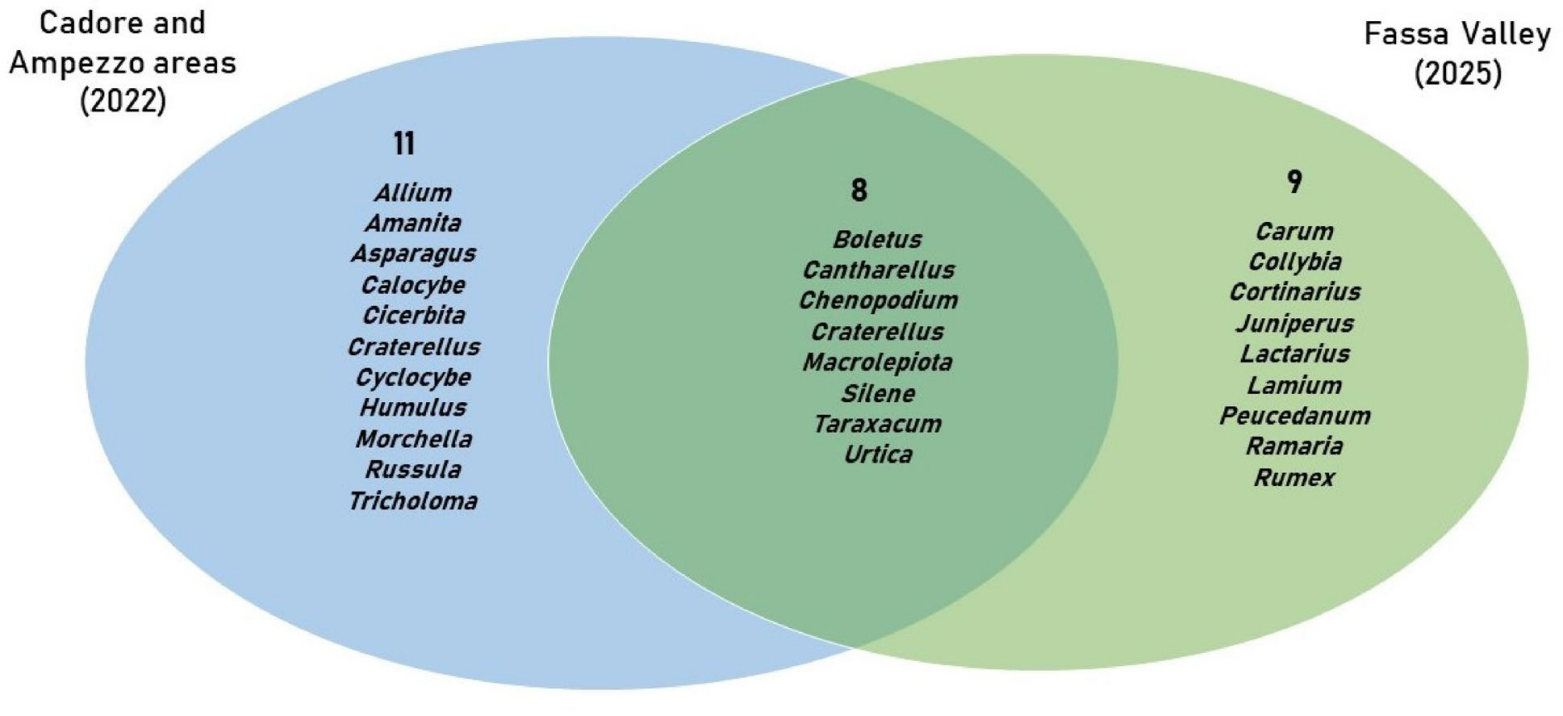
Venn Diagram showing the overlaps between the Ladin wild plant genera reported as locally used as wild vegetables, seasoning, and mushrooms in Cadore and Ampezzo areas and in the Fassa Valley

The Jaccard similarity index between 1980s and 2020s Ladin data is 0.46, suggesting a moderate degree of continuity (Fig. 5). The cross-spatial comparison among Fassa, Cadore, and Ampezzo (Figs. 6 and 7) revealed a heterogeneous pattern of knowledge retention, with the Fassa Valley displaying the highest diversity and internal consistency (ICF = 0.82) compared to other sites.

These results suggest that language vitality may contribute to maintaining ethnobotanical knowledge diversity in specific contexts, although this relationship is not consistent across all domains of practice, with areas where Ladin is still spoken daily showing higher cultural coherence and knowledge diversity.

## Discussion

### Decline of local ecological knowledge in Alpine communities

This study highlights a paradox in biocultural diversity: the persistence of minority languages does not automatically ensure the continuity of local ecological knowledge (LEK). In the Cimbrian, Mòcheno, and Ladin valleys of Northern Italy, our field observations and results indicate that, although these minority languages are reported to remain in use and are, in some cases, supported through school programs, the actual repertoire of wild plant use in daily life has significantly narrowed compared to records from the 1980s [48]. This pattern is supported by the observed reduction in taxa (e.g., a 65% decline in the Mòcheno community) and lower consensus values, indicating fragmentation of shared knowledge.

Particularly in Lusern and the Fassa Valley, and to a lesser extent in Bernstol, comprehensive programs for safeguarding the local language have been implemented. These initiatives include the teaching of local nature vocabulary in schools, museum exhibitions, and community publications. Institutional initiatives (e.g., exhibitions and educational programs) support linguistic and cultural visibility but do not necessarily sustain everyday ecological practice.

Despite the continued use of minority languages, many wild species that were once central to household herbal repertoires, such as *Achillea millefolium*, *Artemisia absinthium*, and *Sambucus nigra* are no longer gathered. Contemporary use has shifted toward a more restricted set of species, particularly for herbal teas, while data on wild vegetables, fruits, and mushrooms are limited due to the lack of systematic field recording over recent decades [4].

In parallel, elements of wild plant and mushroom knowledge increasingly persist through processes of cultural representation rather than daily practice. Exhibitions, publications, and the commercialisation of herbal products aimed at visitors contribute to the visibility of local ecological knowledge but often detach it from its original subsistence and care-based contexts. This form of cultural heritagisation preserves terminologies, symbols, and selected practices while narrowing the broader repertoire of everyday ecological engagement. Such processes do not necessarily imply a loss of cultural value; however, they transform LEK from a lived system of practice. In this sense, the persistence of linguistic and symbolic references to wild plants may coexist with the erosion of embodied skills, reinforcing the disjunction between knowing about nature and knowing through sustained interaction with local ecosystems. Similar dynamics have been observed in contemporary gastronomic contexts, where wild plants are reintroduced through foraging-centred restaurants as innovative or identity-bearing ingredients, often detached from local subsistence traditions and intergenerational knowledge transmission [54].

These findings suggest that language resilience preserves terminologies, categories, and symbolic references but does not necessarily guarantee the enactment of the full spectrum of foraging practices. Minority languages can remain robust and continue to transmit ecological vocabulary, yet the everyday collection and use of wild food plants and mushrooms often decline when traditional livelihoods change or become less central to daily life [5]. The persistence of linguistic knowledge, therefore, does not automatically translate into the retention of embodied ecological practices, which rely on viable smallholder economies, access to functional landscapes, and the integration of agro-pastoral routines into community life [9, 20, 49]. The observed pattern is therefore not a uniform erosion of local ecological knowledge, but a process of selective resilience, in which forest-based practices embedded in commons persist, while herb- and farm-dependent knowledge collapses alongside agro-pastoral livelihoods.

### Collapse of small-scale farming and the rise of (over)tourism as structural drivers of LEK erosion

Tourism-driven economic restructuring has not merely replaced small-scale farming as a source of income in Alpine valleys but has profoundly altered the temporal, spatial, and social conditions under which local ecological knowledge (LEK) was historically produced and transmitted. The shift from agro-pastoral livelihoods to service-oriented economies has reorganised daily rhythms, labour allocation, and patterns of landscape access, reducing opportunities for sustained, embodied engagement with pastures, forest margins, and field edges where wild food plants and mushrooms were traditionally encountered and learned.

By concentrating labour in hospitality, transport, and seasonal services, tourism diminishes the time and necessity for subsistence-oriented practices, while simultaneously reshaping landscapes through infrastructure development, recreational zoning, and intensified human presence. As a result, the experiential contexts required for intergenerational learning, routine grazing, haymaking, fuelwood collection, and daily foraging, are progressively eroded. Linguistic competence may persist, allowing younger generations to name plants and fungi, yet without regular practice embedded in everyday livelihoods, such knowledge remains largely symbolic rather than enacted.

The decisive factor in the observed erosion of local ecological knowledge (LEK) in the Alpine valleys appears to be the decline of small-scale farming. Traditionally, farms provided both ecological niches, such as grazing pastures, hay meadows, and orchard margins, and socio-economic arenas in which the collection and use of wild plants were embedded in daily subsistence. As smallholder agriculture diminished, opportunities for experiential learning and the transmission of embodied ecological knowledge also declined, decoupling linguistic competence from the practical skills required to forage, process, and utilise wild food plants and mushrooms [41].

During fieldwork in the Fassa Valley, only a few malghe (summer Alpine huts) still hosted livestock, while many pastures were abandoned. As farming households disappeared and overtourism expanded in the past two decades, younger generations retained the ability to name plants but had limited opportunities to practice their use in everyday agro-pastoral routines. This observation aligns with findings from other contexts, where shifts in livelihood structures, rather than language vitality per se, drive the decline of LEK [37, 38].

As Ross [47] noted, linguistic categories can outlive the practices that gave them meaning, functioning as “empty shells” of cultural memory. In this sense, cultural continuity at the linguistic level can persist even amid ecological discontinuity, where words and plant names remain, but their practical enactment in daily life fades. The decline of small-scale farming has thus reshaped the Alpine landscape from one of subsistence and agro-pastoral resilience toward reduced engagement with wild plant and mushroom resources. Even as resident populations remain stable, the loss of farm-based livelihoods constrains opportunities for embodied ecological knowledge, weakening the intergenerational transmission of practices tied to local ecosystems. This pattern underscores the crucial role of smallholder agriculture in sustaining LEK, highlighting that language competence alone is insufficient to preserve ecological practices [9, 20, 49].

### Commons as anchors of biocultural resilience

The persistence of specific foraging practices in the studied Alpine valleys is closely linked to the continued functioning of *usi civici*. customary commons regimes that regulate collective access to forests and pastures. These institutions constitute a form of socio-ecological infrastructure that mediates relationships between people, landscapes, and resources, enabling continuity of practice even as other subsistence domains, such as small-scale farming, have declined. Rather than representing residual or archaic land-tenure systems, *usi civici* operate as adaptive governance arrangements that structure access, responsibility, and intergenerational learning within shared ecological spaces. Drawing on the frameworks of commons theory and adaptive co-management, such regimes support not only sustainable resource use but also the transmission of ecological knowledge through repeated, embodied interaction with forest environments. By maintaining communal access and shared stewardship, *usi civici* preserve the conditions under which knowledge of wild food plants and mushrooms can remain practiced, monitored, and socially embedded, buffering LEK against the fragmenting effects of market integration and livelihood transformation.

Specific forms of ecological engagement have persisted in Alpine valleys thanks to the *usi civici*, customary commons regimes that regulate collective access to forests and pastures. Across all study areas, Lusern, Bernstol/Mòcheni Valley, Cadore, Ampezzano, and the Fassa Valley, these institutions have structured community interactions with forest ecosystems. Although their scope and enforcement differ, they collectively ensure that woodlands and pastures remain under communal rather than private control.

In Lusern and the Mòcheni Valley, communal property registers (*catasti delle proprietà collettive*) continue to regulate forest access and the gathering of non-timber products through assemblies and local forest guards. In Cadore and Ampezzo, regole and vicinie, ancient forms of Alpine self-governance, maintain shared management of forests, balancing small-scale timber extraction with subsistence foraging. In the Fassa Valley, *usi civici* under the jurisdiction of the *Comun General de Fascia* safeguard access to high-elevation forests and berry-rich pastures, ensuring continued engagement with these resources. Examples of this engagement are visible in local restaurants, where seasonal mushroom dishes and homemade herb grappa celebrate communal use of forest products.

The enduring presence of these *usi civici* represents critical socio-ecological infrastructure that sustains the resilience of foraging practices in Alpine communities. Drawing on the frameworks of Ostrom [34] and Berkes ([10]; Common property resources [16]), commons are not mere historical remnants but adaptive institutions that regulate resource use, maintain ecological integrity, and foster social learning. In Alpine contexts, communal governance has historically underpinned both environmental sustainability and cultural continuity.

In Lusern, for example, communal access supports regulated mushroom and berry gathering. Practices are grounded in reciprocity and respect, reflecting Ostrom’s principles of robust common-pool management. Families teach sustainable harvesting methods, such as avoiding knives and restoring disturbed soil, linking ecological ethics with social norms.

Similarly, in the Fassa Valley, families continue to exercise communal rights to gather mushrooms (*Boletus*, *Cantharellus*) and berries (*Vaccinium myrtillus*, *V. vitis-idaea*). From a resilience perspective, these activities act as feedback mechanisms that couple cultural memory with ecological renewal, enabling adaptive co-management within trusted community frameworks.

The contrasting trajectories observed between declining herbal practices and the resilience of mushroom and wild fruit gathering underscore the importance of access regimes and livelihood contexts in shaping LEK persistence. While the loss of farming and household-based production has undermined the everyday use of synanthropic and meadow species traditionally associated with teas, remedies, and domestic preparations, forest-based resources remain embedded within communal governance systems that facilitate continued engagement. Under these conditions, foraging for mushrooms and berries functions not merely as a recreational activity but as a culturally regulated practice supported by shared norms, access rights, and ecological monitoring. This asymmetry highlights that resilience within biocultural systems is uneven and domain-specific, shaped less by language vitality alone than by the institutional and ecological contexts that enable knowledge to be enacted.

The *usi civici* thus functions as a reservoir of biocultural resilience, integrating ecological, social, and cognitive dimensions. Unlike privatised or state-managed forestry, they maintain the connective tissue between people and ecosystems, sustaining what Berkes [11] describes as “knowledge–practice–belief complexes.” Even amid the decline of small-scale farming, forest-based foraging endures as a living, adaptive tradition. Communal property systems provide institutional continuity that anchors ecological engagement within shifting economies. They prevent forest alienation and buffer against the fragmentation of local ecological knowledge observed elsewhere. Under these conditions, foraging continues as both a cultural practice and an ecological monitoring tool, linking community well-being with biodiversity. Comparable dynamics have been documented in other European contexts, where informal, community-based food systems function as biocultural refugia that sustain ecological knowledge, social cohesion, and small-scale economies amid structural transformations [40].

The resilience of wild fruit and mushroom foraging is thus not an accidental survival, but an outcome of governance aligned with adaptive co-management and polycentric principles [26, 34]. Alpine *usi civici* exemplifies how commons can serve as living laboratories for sustainable transitions, where knowledge, shared responsibility, and ecological reciprocity converge to sustain both biodiversity and cultural identity. Strengthening and recognising these communal systems should be central to biodiversity conservation and resilience-based management in European mountain regions.

Finally, wild plant- and mushroom-based products derived from local LEK, coupled with interpretive narratives that communicate their cultural and ecological significance, can support both economic value and the continuity of Alpine biocultural heritage.

While *usi civici* emerge as key infrastructures supporting the persistence of forest-based foraging, they are not immune to internal transformations, generational disengagement, or bureaucratic constraints. Their resilience depends on continued community participation, legal recognition, and adaptive governance. The persistence of mushroom and berry gathering should therefore not be interpreted as guaranteed continuity, but as a contingent outcome shaped by access, social norms, and broader political-economic conditions. This study does not include a direct quantitative assessment of linguistic vitality (e.g., speaker frequency or intergenerational transmission rates). Therefore, references to language persistence are based on contextual observations rather than primary linguistic data. The interpretation of these findings requires caution in light of several methodological constraints. The diachronic comparison between contemporary and historical datasets is inherently limited by differences in research design, sampling intensity, and thematic focus, as earlier studies primarily documented medicinal plants and did not systematically record fungal uses. Consequently, the comparison is indicative of general trends rather than directly comparable quantitative measures. In addition, the use of purposive and snowball sampling may have favoured the inclusion of participants with stronger ecological knowledge, potentially affecting the representativeness of the sample. Variations in data collection across study areas, particularly in the categories of plant uses recorded, further limit cross-site comparability. The reliance on participants’ recollections of past practices also introduces the possibility of recall bias. Finally, although minority languages are discussed as part of the broader biocultural context, no systematic assessment of linguistic vitality or intergenerational transmission was conducted; therefore, any inference regarding language persistence remains interpretative rather than empirically grounded.

## Conclusion

This study provides new evidence that the persistence of minority languages does not necessarily correspond to the maintenance of local ecological knowledge. In the Cimbrian, Mòcheno, and Ladin-speaking Alpine areas, minority languages remain actively transmitted through schools, media, and cultural initiatives, yet the practical repertoire of wild plant use, particularly for teas and homemade liquors, has declined significantly. This divergence highlights a paradox: while vernacular terminologies survive, the embodied skills and daily practices associated with them have eroded, mainly due to the collapse of small-scale farming and the transformation of livelihoods.

At the same time, specific practices, such as mushroom and wild fruit gathering, persist thanks to collective land-use institutions (*usi civici*), which provide socio-ecological infrastructure for intergenerational learning and sustainable resource management. These commons act as reservoirs of resilience, maintaining ecological engagement even amid broader socio-economic change.

The findings underscore that protecting biocultural heritage requires more than language revitalisation: it demands the integration of ecological and economic frameworks that support traditional knowledge, including smallholder agriculture, sustainable forest governance, and community-based stewardship. Only by reconnecting language, livelihood, and landscape can Alpine communities maintain both cultural and ecological diversity.

Socio-ecological transitions driven by tourism, market integration, and agricultural decline can decouple language from practice, leading to the loss of embodied ecological knowledge. Future research should adopt longitudinal and comparative approaches to monitor LEK transformations, examine contextual socio-economic and political drivers, and evaluate the role of commons in sustaining biocultural continuity. Alpine ethnobotany thus provides a lens for understanding broader sustainability challenges, showing that the resilience of human knowledge depends on the strength of the ecologies that sustain it.

## Data Availability

Data supporting the reported results are presented in the manuscript. Further inquiries can be directed to the corresponding author.

## Abbreviations

LEK: Local Ecological Knowledge
UV: Use Value
ICF: Informant Consensus Factor
CI: Cultural Importance Index
WFPMs: Wild Food Plants and Mushrooms
